# Nuclear CaMKII Isoforms as Regulators of Transcription: From Developmental to Pathological Persistence

**DOI:** 10.3390/medsci13040246

**Published:** 2025-10-27

**Authors:** Areli Marlene Gaytán-Gómez, Claudio Adrián Ramos-Cortés, Ricardo Xopan Suarez-García, Diego Alberto Martínez-Islas, Axel Tonatiuh Marroquin-Aguilar, Fernanda Avelino-Vivas, Dafne Montserrat Solis-Galván, Alexis Arturo Laguna-González, Bruno Manuel García-García, Eduardo Minaya-Pérez, Efren Quiñones-Lara, Axel Eduardo Muciño-Galicia, Olga Villamar-Cruz, Luis Enrique Arias-Romero, Sonia León-Cabrera, Leonel Armas-López, Héctor Iván Saldívar-Cerón

**Affiliations:** 1 Unidad de Remisión de Diabetes Mellitus (URDM), Facultad de Estudios Superiores-Iztacala, Universidad Nacional Autónoma de México, Tlalnepantla 54090, Mexico; marlenegaytangmz@gmail.com (A.M.G.-G.); claussramosone@gmail.com (C.A.R.-C.); ricardoxopan@comunidad.unam.mx (R.X.S.-G.); diegomartinezislas411@comunidad.unam.mx (D.A.M.-I.); axelnrd@comunidad.unam.mx (A.T.M.-A.); f_avelinovivas@comunidad.unam.mx (F.A.-V.); dafnesg2@comunidad.unam.mx (D.M.S.-G.); alexis_lag@comunidad.unam.mx (A.A.L.-G.); bruno.garcia12@comunidad.unam.mx (B.M.G.-G.); eduardo_minaya@comunidad.unam.mx (E.M.-P.); 2 Carrera de Médico Cirujano, Facultad de Estudios Superiores-Iztacala, Universidad Nacional Autónoma de México, Tlalnepantla 54090, Mexico; 317075993@iztacala.unam.mx; 3 Laboratorio de Medicina de la Conservación, Escuela Superior de Medicina, Instituto Politécnico Nacional (IPN), Mexico City 11350, Mexico; 4 Departamento de Biomedicina Molecular, Centro de Investigación y de Estudios Avanzados del Instituto Politécnico Nacional (CINVESTAV-IPN), Ciudad de Mexico 07360, Mexico; efren.quinones@cinvestav.mx; 5 Unidad de Biomedicina (UBIMED), Facultad de Estudios Superiores Iztacala, Universidad Nacional Autónoma de México, Tlalnepantla 54090, Mexico; olga.villamar@unam.mx (O.V.-C.); larias@unam.mx (L.E.A.-R.); soleonca@iztacala.unam.mx (S.L.-C.); larmas@unam.mx (L.A.-L.)

**Keywords:** calcium-calmodulin-dependent protein kinase II, gene expression regulation, alternative splicing, cell nucleus, transcription, genetic

## Abstract

Calcium/calmodulin-dependent protein kinase II (CaMKII) comprises multiple isoforms with distinct nuclear variants that exert transcriptional control in a context-dependent manner. Among them, CaMKIIδB and δ9 in the heart, and CaMKIIγ in the nervous system, have emerged as regulators of chromatin dynamics, transcription factor activity, and developmental gene programs. Nuclear localization is driven by splice-dependent nuclear localization sequences, with phosphorylation at defined serine residues modulating import and retention. Evidence supports CaMKII-dependent phosphorylation of class IIa HDACs (Ser467/Ser632 in HDAC4), linking CaMKII to MEF2 activation in cardiac hypertrophy, and interactions with NF-κB and HSF1 further expand its nuclear repertoire. In the nervous system, CaMKIIγ contributes to kinase-dependent gene expression, potentially influencing plasticity and disease susceptibility. While these mechanisms highlight nuclear CaMKII as an isoform-specific regulator of transcription, direct evidence remains elusive, and several CaMKII putative substrates require further validation. This review synthesizes current knowledge on nuclear CaMKII isoforms, emphasizes established mechanistic pathways, and outlines unsolved questions critical for understanding their roles in development, disease progression, and therapeutic targeting.

## 1. Introduction

Calcium/calmodulin-dependent protein kinase II (CaMKII) is more than a cytosolic decoder of calcium transients; its nuclear isoforms embody a molecular bridge between developmental gene encoding and the persistence of pathological programs. Over the last three decades, splicing variants with nuclear localization sequences, including CaMKIIδB, δ9, and CaMKIIγ, have emerged as central players in transcriptional regulation [1,2,3,4]. Within the nucleus, these isoforms phosphorylate class IIa histone deacetylases (HDAC4 at Ser467/632), enabling MEF2-dependent transcription [5] and interact with NF-κB and HSF1 to modulate stress adaptation [6,7,8]. Additional evidence links nuclear CaMKII to chromatin modifications, including histone H3 phosphorylation at Ser10 under hemodynamic stress [9]. Together, these findings highlight an isoform-specific layer of CaMKII signaling that coordinates transcriptional responses across development and disease. Yet, despite compelling evidence, isoform-dependent mechanisms remain incompletely defined, and several proposed phosphorylation targets require further validation [10,11,12]. This review seeks to consolidate current insights, distinguish established mechanisms from speculative models, and chart the unresolved questions that will define the next phase of CaMKII nuclear biology. To ensure comprehensive coverage, we conducted a structured literature search in PubMed, Scopus, and Web of Science databases, encompassing publications from 1990 to September 2025. The following Boolean query was used: (“CaMKII” OR “Calcium/calmodulin-dependent protein kinase II”) AND (“nucleus” OR “nuclear localization” OR “transcription” OR “chromatin” OR “isoform” OR “δB” OR “δC” OR “γ”). Articles were included if they (i) provided mechanistic or functional evidence on nuclear CaMKII isoforms, (ii) described isoform-specific localization or signaling, and (iii) were published in peer-reviewed journals. Reviews lacking primary mechanistic data and studies confined exclusively to cytosolic functions were excluded. When possible, the source of evidence was annotated throughout the manuscript—indicating whether findings originated from cellular, animal, or clinical models—to improve interpretive transparency. This review integrates experimental and conceptual advances published up to 2024, offering a unified view of nuclear CaMKII isoforms as spatiotemporal regulators of transcription. By linking developmental, homeostatic, and pathological contexts, it proposes a novel synthesis in which nuclear localization operates as a regulatory layer of transcriptional memory.

## 2. Nuclear Trafficking Logic: Isoforms, Assembly, and CaM Shuttling

Nuclear access is not automatic for CaMKII. It emerges from three convergent layers: alternative splicing that installs an import-competent NLS (KKRK) in specific variants, post-translational gating that masks or unmasks this signal, and the multimeric context of the dodecameric holoenzyme [2,3,4,13,14,15,16]. While cytoplasmic functions of CaMKII are well established, nuclear presence demands both a permissive isoform design and a cellular state that favors import—linking calcium dynamics, NLS phosphorylation status, and holoenzyme composition [12,17,18]. This section dissects those prerequisites: which splice variants acquire nuclear entry, how phospho-switches gate import, and how assembly logic enables co-transport of subunits and, ultimately, CaM shuttling to nuclear effectors [19].

### 2.1. Nuclear Localization by Design: The NLS Motif and Its Control

**Figure 1:**
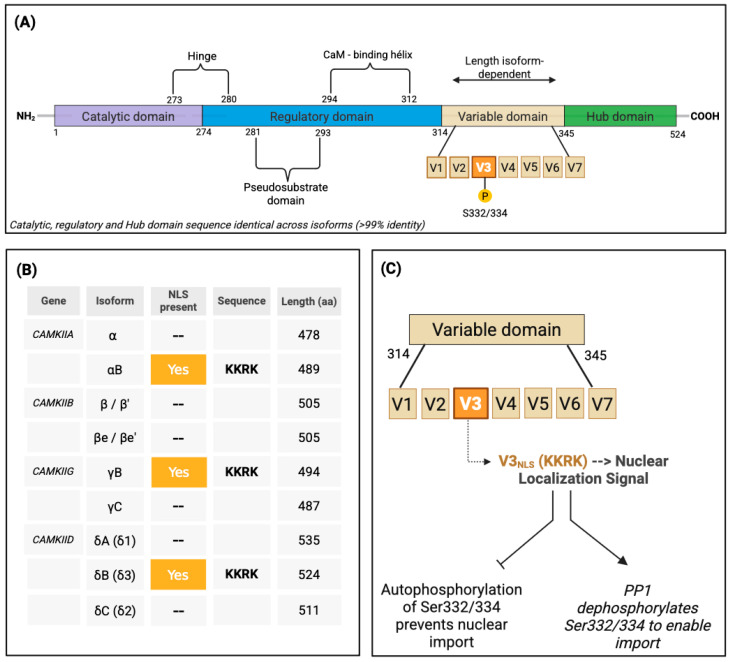
Isoform-Specific Nuclear Import of CaMKII Is Regulated by Alternative Splicing and a Phosphorylation-Gated NLS Switch. (**A**) Domain architecture of CaMKII showing the conserved catalytic, regulatory, variable, and hub regions. Nuclear import is governed by isoform-dependent splicing of variable exons (V1–V7), with V3 encoding a nuclear localization signal (KKRK) in select splice variants. (**B**) Human CaMKII isoforms showing presence or absence of V3^NLS and corresponding sequence data. Only αB, γB, and δB incorporate the KKRK motif and are predicted to localize to the nucleus. (**C**) Schematic of the regulatory logic of nuclear entry. The V3^NLS motif enables import, but autophosphorylation at Ser332 (δB) or Ser334 (γB) prevents nuclear translocation. Dephosphorylation by PP1 restores nuclear access. This dynamic switch integrates calcium signals with transcriptional access via compartmentalized gating.

Unlike transcription factors or chromatin kinases that carry prototypical NLS motifs, most CaMKII isoforms lack canonical import sequences [18,20]. This is particularly true for α and β, which dominate in neurons and remain excluded from the nucleus under basal conditions. These isoforms are effectively non-nuclear unless recruited by association. In contrast, specific splice variants of γ and δ isoforms incorporate a positively charged motif (KKRK) encoded by alternative exons. This NLS, best characterized in CaMKIIδB (δ3), also appears in γB and αB, indicating a conserved strategy of regulated nuclear targeting across the CaMKII family [2,3,15,21,22]. Yet the presence of an NLS is not sufficient for import. Phosphorylation adjacent to the motif—particularly at Ser332 (δB) or Ser334 (γB)—masks the import signal and prevents nuclear entry [13,15]. Kinases such as CaMKI and CaMKIV act as gatekeepers, while dephosphorylation by protein phosphatase 1 (PP1) restores access [13,15,23,24]. This phospho-switch integrates calcium oscillations with transcriptional gating, making localization—not just activation—a defining aspect of CaMKII function (Figure 1) [17].

### 2.2. Assembly Dictates Destiny: The Holoenzyme as a Nuclear Unit

**Figure 2:**
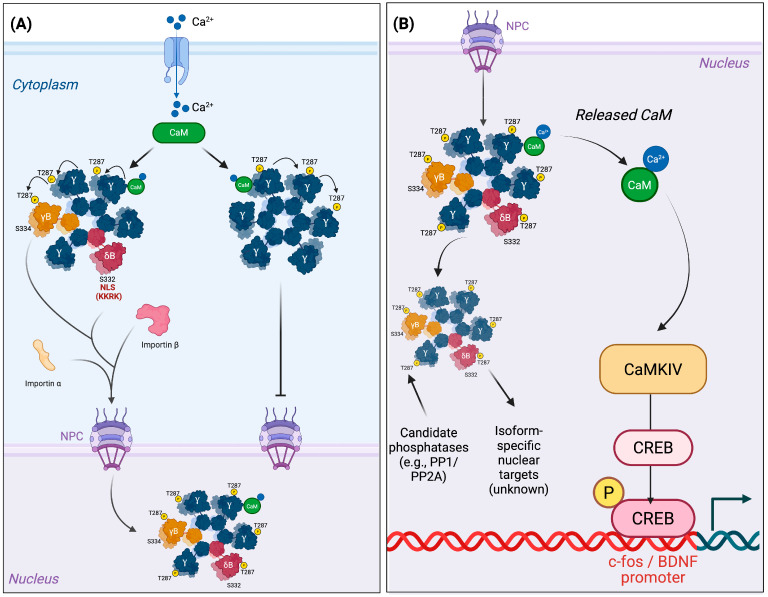
Isoform-specific logic of CaMKII nuclear import and CaM shuttling. (**A**) Ca^2+^ influx activates CaMKII holoenzymes, promoting T287 autophosphorylation and calmodulin (CaM) trapping. Holoenzymes containing NLS-bearing subunits (γB, δB) interact with importin α/β and undergo nuclear import through the nuclear pore complex (NPC), whereas assemblies composed exclusively of non-NLS isoforms remain cytosolic. Nuclear entry is gated by phosphorylation of Ser332 (δB) or Ser334 (γB), which prevents import, and dephosphorylation by candidate phosphatases (e.g., PP1, PP2A) that restores access. (**B**) Once inside the nucleus, holoenzymes may release CaM–Ca^2+^, which activates CaMKIV, leading to CREB phosphorylation and transcription of activity-dependent genes (e.g., c-fos, BDNF). Additional nuclear functions of CaMKII holoenzymes remain incompletely defined, suggesting isoform-specific transcriptional or epigenetic roles yet to be characterized. Subunits labeled “Y” represent isoforms lacking an NLS (α, β, δC, etc.).

CaMKII functions not as monomers but as dodecameric holoenzymes composed of mixed subunits [4,16]. In cells where multiple isoforms are co-expressed—such as neurons and cardiomyocytes—assemblies may include both NLS-bearing (e.g., δB, γB) and NLS-lacking (e.g., δC, α, β) monomers. Nuclear access is therefore governed not by the identity of a single subunit but by the stoichiometry of the entire complex [2,14,15]. Imaging studies indicate that incorporation of a few NLS-positive subunits can promote nuclear import under specific activation states of the holoenzyme, enabling non-nuclear isoforms to enter as passive co-passengers [15]. This cooperative property of the multimeric complex is illustrated in Figure 2. An important mechanistic question arises: does nuclear translocation merely redistribute cytosolic kinase capacity, or does it provide access to specialized nuclear signaling? Available evidence favors the latter, as nuclear import allows isoform-specific transcriptional regulation inaccessible to cytosolic assemblies [5,16,25,26]. In this view, holoenzyme composition operates as an internal integrator of isoform expression, cellular state, and spatial fate.

### 2.3. Nuclear Export and Dampening Mechanisms of CaMKII

Beyond nuclear import, several mechanisms restrict the residence time of CaMKII within the nucleus. For δ3/δB isoforms, Ser332 operates as a reversible switch: PP1-mediated dephosphorylation unblocks the nuclear localization signal (NLS) and favors import, whereas phosphorylation at Ser332 enhances 14-3-3 binding, masks the NLS, and promotes cytosolic retention or export. This creates a bidirectional shuttle wherein CaMKII transitions between nucleus and cytoplasm according to stimulus duration (PP1 ←→ 14-3-3). In parallel, CRM1/exportin-1 mediates the nuclear export of several CaMKII-regulated cargoes such as HDAC4, thereby attenuating transcriptional signaling once stress cues subside. Moreover, Thr253, a bona fide autophosphorylation site that influences molecular targeting without altering catalytic activity, modulates CaMKII subcellular anchoring. Although direct evidence in cardiac tissue remains limited, phospho-T253 likely stabilizes cytosolic or postsynaptic density anchoring, indirectly reducing nuclear residency, while the dephosphorylated form enlarges the pool available for nuclear import. Collectively, Ser332/PP1–14-3-3 cycling, CRM1-dependent export, and T253-dependent localization constitute an integrated dampening layer that constrains the persistence of nuclear CaMKII signaling [5,12,13,15,27,28,29].

## 3. Nuclear Functions in Physiology: Isoform-Specific Signaling Beyond the Cytosol

Once in the nucleus, CaMKII is not merely a generic decoder of calcium transients; it acts as an isoform- and context-specific regulator of transcriptional programs, chromatin accessibility, and cell identity. In cardiomyocytes, nuclear CaMKIIδB phosphorylates class IIa HDACs to derepress MEF2-dependent genes, while CaMKII also marks chromatin directly (H3-Ser10) under hemodynamic stress [5,9]. In neurons, CaMKIIγ ferries Ca^2+^/calmodulin into the nucleus to engage the CaMKIV–CREB axis and drive immediate-early gene expression [19,28]. By contrast, CaMKIIδ9 preferentially amplifies NF-κB signaling during injury, illustrating pathway selectivity across splice variants [6,7]. These examples underscore that nuclear functions vary by isoform, cell type, developmental stage, and disease state, reflecting a convergence of structural specialization, post-translational gating, and spatial logic [24,25,26].

### 3.1. Transcriptional Decoding: CREB, Coactivators, and Immediate Early Genes

CaMKII is not a transcription factor, yet specific isoforms operate as transcriptional gatekeepers. The best-characterized example is CaMKIIγ, whose nuclear function relies not on DNA binding but on its ability to shuttle active Ca^2+^/calmodulin (CaM) into the nucleus [19]. Synaptic activation or depolarization triggers CaMKIIγ autophosphorylation at Thr287, locking CaM into a high-affinity complex that is imported via the isoform’s intrinsic nuclear localization signal (NLS). Once inside, CaM activates CaMKIV, leading to CREB phosphorylation at Ser133, recruitment of CBP, and induction of immediate early genes (IEGs) such as c-fos, Arc, and BDNF (rat hippocampal neurons) [19,30]. This cascade represents the molecular bridge between synaptic activity and nuclear gene expression, as disruption of CaMKIIγ’s NLS or its genetic deletion abolishes CREB activation despite intact cytosolic Ca^2+^ signals [12]. This function is isoform-specific. Cytosolic CaMKIIα and β dominate dendritic compartments and support local plasticity but do not access the nucleus. Their deletion impairs synaptic transmission but not calcium-responsive transcription [31]. Conversely, CaMKIIγ is dispensable for dendritic propagation but essential for nuclear engagement, illustrating a division of labor among isoforms [28]. Together with CaMKIV, CaMKIIγ establishes a multi-tiered decoding system that converts transient Ca^2+^ influx into durable transcriptional programs. Beyond physiology, CaMKIIγ dysfunction has pathological implications. Mutations in *CAMK2G* cause neurodevelopmental disorders with impaired nuclear targeting [32], while variants in *CAMK2A/B* disrupt synaptic transcriptional coupling [33]. Such evidence suggests that inability to convert neuronal activity into lasting genomic responses may contribute to cognitive deficits in conditions including Alzheimer’s disease, schizophrenia, and Rett syndrome [34]. In summary, CaMKIIγ does not merely decode calcium signals—it imprints them onto the genome, establishing transcriptional memory that outlasts the ion transient and anchors long-term plasticity.

### 3.2. Modulation of Transcription Factors: Precision Tuning by Nuclear CaMKII

**Figure 3:**
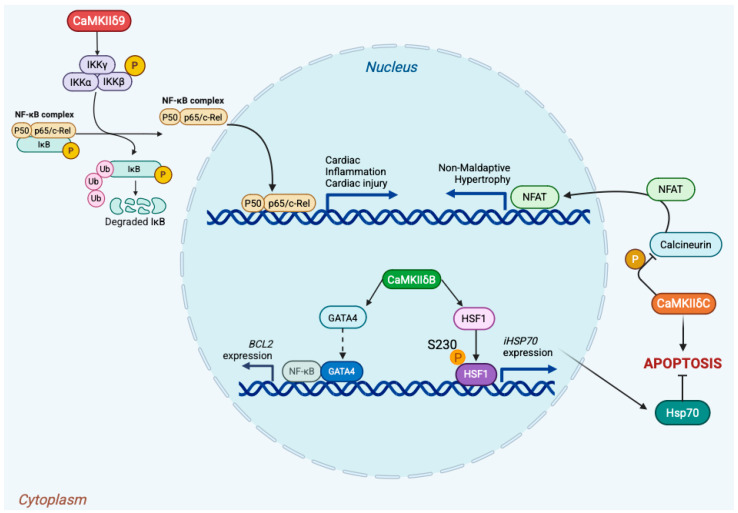
Isoform-specific nuclear targets of CaMKII in cardiomyocytes. CaMKIIδ9 activates the NF-κB pathway by phosphorylating the IKK complex, leading to IκB degradation and p65/p50 nuclear translocation, thereby promoting inflammatory and injury-responsive gene expression [6,7]. In parallel, nuclear CaMKIIδB phosphorylates transcriptional regulators: it enhances GATA4-dependent activation of the BCL2 promoter and modifies HSF1 at Ser230, inducing iHSP70 expression and cytoprotection [37,38] By contrast, cytosolic CaMKIIδC phosphorylates calcineurin A, restraining NFAT nuclear translocation and thereby antagonizing maladaptive hypertrophy [40] Together, these isoform-specific programs couple calcium signals to pro-survival, inflammatory, and anti-hypertrophic transcriptional outputs in the stressed heart.

Nuclear CaMKII isoforms not only relay calcium signals but also remodel transcriptional circuitry through direct post-translational modification of transcription factors and cofactors. This control is highly context-dependent, determined by isoform identity, subcellular localization, and cellular state. In the adult heart, CaMKIIδ splice variants illustrate this logic: δB is enriched in the nucleus, whereas δC (historically δ2) is predominantly cytosolic; α/β isoforms are minimally expressed in myocardium [35,36]. In cardiomyocytes, nuclear CaMKIIδB promotes cytoprotective transcription. It is required for GATA4-dependent activation of the BCL2 promoter and robust recruitment of GATA4 to its −266 site (in vivo mouse myocardium), thereby sustaining anti-apoptotic gene expression [37]. Independently, δB phosphorylates HSF1 at Ser230, enhancing its transactivation potential and inducing iHSP70 (neonatal rat cardiomyocytes), a program that confers resistance to ischemia/reperfusion and proteotoxic stress [38,39]. Collectively, these pathways place δB upstream of pro-survival transcription driven by GATA4 and HSF1. By contrast, inflammatory and proliferative transcription via NF-κB has been linked to CaMKIIδ9. In human and rodent cardiomyocytes, δ9 interacts with the IKKβ/IκBα complex, promoting IκBα phosphorylation, p65 nuclear translocation, and activation of NF-κB target genes during reperfusion injury [6,7]. These observations highlight isoform-selective access to the NF-κB axis in the heart. CaMKII also restrains pathological gene programs by antagonizing calcineurin–NFAT signaling. Cytosolic CaMKIIδC phosphorylates calcineurin A at Ser411, reducing its phosphatase activity, thereby increasing NFAT phosphorylation and limiting its nuclear accumulation [40]. Beyond transcription factors, nuclear CaMKII regulates chromatin-level control. CaMKIIδ phosphorylates class IIa HDAC4 (Ser467/Ser632), promoting 14-3-3 docking and nuclear export, which derepresses MEF2-dependent transcription [5]. Additionally, CaMKIIδ has been shown to phosphorylate histone H3 at Ser10 in stressed myocardium, linking calcium signals to nucleosomal modifications that increase accessibility [9]. Taken together, these mechanisms establish CaMKII isoforms as precision tuners of transcription, acting with spatial and substrate specificity rather than as simple on/off switches. These isoform-specific transcriptional circuits are summarized in Figure 3.

### 3.3. Epigenetic Control via Class IIa HDAC Phosphorylation

**Figure 4:**
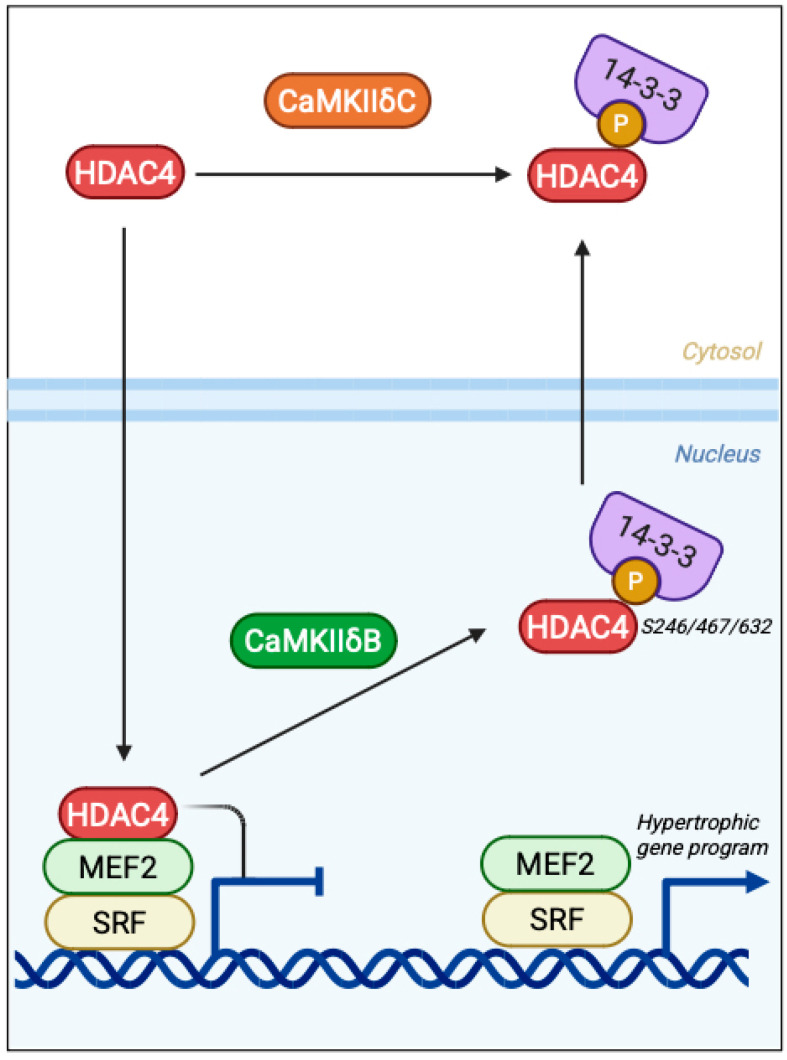
Epigenetic control of transcription by CaMKIIδ isoforms. Nuclear CaMKIIδB phosphorylates HDAC4 at Ser246/467/632, promoting 14-3-3 binding, nuclear export, and derepression of MEF2/SRF-dependent hypertrophic gene expression. In contrast, cytosolic CaMKIIδC limits sustained nuclear access to HDAC4, constraining chromatin-level remodeling.

Nuclear CaMKII reshapes the epigenetic landscape by targeting class IIa histone deacetylases (HDACs), transcriptional repressors that gate access to MEF2-dependent programs. Among these, HDAC4 is the best-characterized substrate in excitable tissues: it binds MEF2 at promoter-proximal chromatin and enforces repression of genes involved in growth, differentiation, and adaptive remodeling [5,41,42]. CaMKIIδB interacts with HDAC4 through a dedicated docking site and phosphorylates it at Ser246, Ser467, and Ser632, creating 14-3-3 binding motifs that mask the NLS and expose the NES. This promotes nuclear export of HDAC4 and derepression of MEF2 activity (cultured mouse cardiomyocytes) [5,41,42,43]. In cardiomyocytes, CaMKII activity is required for agonist-induced cytosolic accumulation of HDAC4, and HDAC4 mutants resistant to CaMKII phosphorylation blunt hypertrophic gene expression, establishing the CaMKII→HDAC4→MEF2 axis as a driver of pro-hypertrophic transcription. This mechanism is summarized in Figure 4, highlighting how CaMKIIδB, but not δC, sustains epigenetic remodeling by regulating HDAC4 nuclear export and MEF2/SRF-driven transcription [5].

While HDAC4 is the dominant CaMKII substrate in cardiomyocytes, HDAC5 can also be targeted in a cell-type-restricted manner. In vascular smooth muscle, AngII–GIT1 signaling recruits CaMKII together with PKD to phosphorylate HDAC5 and drive its nuclear export, thereby relieving repression of MEF2-dependent genes; similar PKD-centric pathways have been described in endothelium [44,45].

Isoform identity further tunes this regulation: δB, with its nuclear localization sequence, resides in the nucleus and provides sustained access to chromatin-bound substrates such as HDAC4. By contrast, δC is largely cytosolic, consistent with more transient nuclear encounters [35,36]. This compartmentalization positions δB as the isoform most likely to act as a chromatin-modifying kinase that links Ca^2+^ dynamics to durable transcriptional reprogramming. Beyond HDACs, nuclear CaMKIIδ can directly modify chromatin. For example, phosphorylation of histone H3 at Ser10 during hemodynamic stress has been reported, providing a direct route by which CaMKII contributes to nucleosomal remodeling and transcriptional amplification in stressed myocardium [9].

### 3.4. Cell Cycle Modulation: CaMKII at the Proliferation–Checkpoint Interface

**Figure 5:**
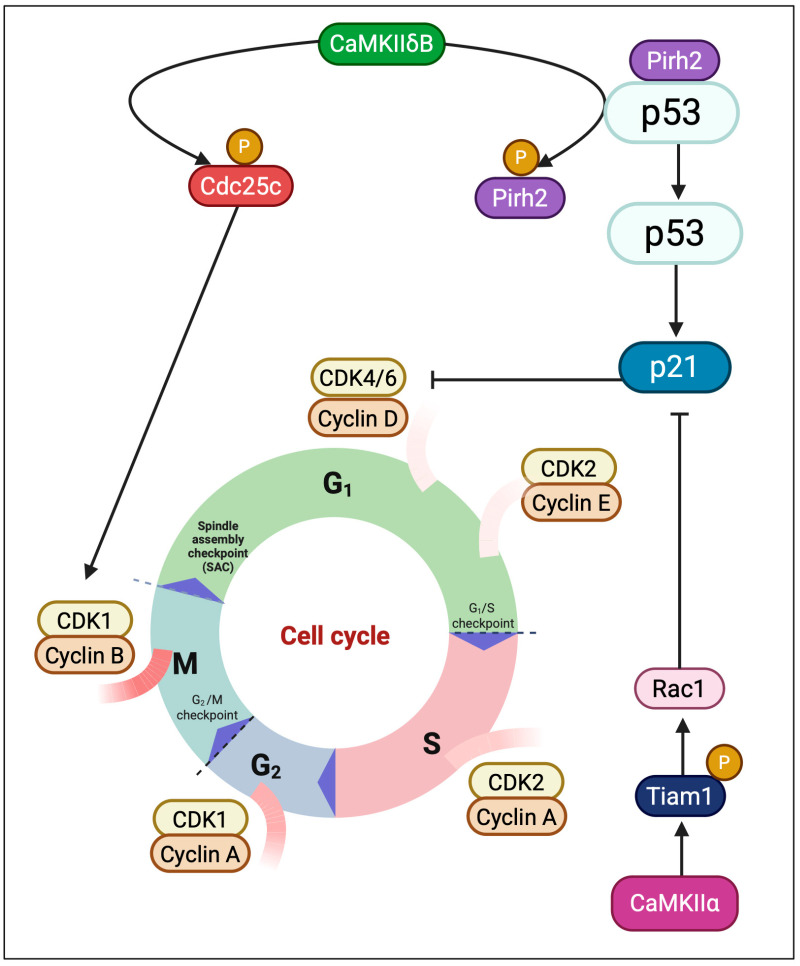
CaMKII isoforms integrate cell-cycle progression and checkpoint control. Nuclear CaMKIIδB phosphorylates Cdc25C, promoting CDK1/Cyclin B activation and mitotic entry, while simultaneously stabilizing p53 via Pirh2 phosphorylation, enhancing p21-mediated checkpoint arrest under stress. In parallel, CaMKIIα facilitates G1/S progression by phosphorylating Tiam1 and activating Rac1, thereby reducing p21 inhibition and enhancing Rb/E2F-dependent transcription control.

Nuclear CaMKII isoforms integrate calcium signaling with cell-cycle control, influencing whether cells progress through division, pause at checkpoints, or undergo differentiation. Evidence from proliferative models suggests that CaMKII activity can support both G1/S and G2/M transitions, although effects are strongly context-dependent and not always isoform-specific. In HeLa cells (in vitro), pharmacologic (KN-93) or peptide (AC3-I) inhibition of CaMKII enforces both G1/S and G2/M arrest, implicating CaMKII in checkpoint release [46,47]. However, these findings rely on inhibitors with known off-target effects, and their interpretation requires caution. In Xenopus egg extracts, CaMKII phosphorylates Cdc25C at Ser287 and delays CDK1–cyclin B activation, underscoring context-dependent outcomes even for the same substrate [48]. Isoform-specific roles are beginning to emerge. In osteosarcoma, nuclear CaMKIIα phosphorylates Tiam1 to activate Rac1, thereby reducing p21^CIP1^, increasing Rb phosphorylation, and promoting G1/S progression; knockdown of CaMKIIα reverses these effects [49]. In epithelial cancers, CaMKII has been linked to NF-κB–driven cyclin D1 expression, but most studies rely on KN-93 and do not definitively assign isoform identity. By contrast, direct activation of IKKβ/p65 by CaMKIIγ has been demonstrated in non–small cell lung cancer (NSCLC cell lines), where γ phosphorylates IKKβ at Ser177/Ser181 to sustain NF-κB activity and proliferation [50,51]. Nuclear CaMKII also contributes to checkpoint fidelity under stress. In oxidative or genotoxic conditions, CaMKII phosphorylates the E3 ligase Pirh2, reducing p53 ubiquitination and stabilizing p53, thereby prolonging checkpoint arrest while damage is resolved [52]. In reproductive biology, CaMKIIγ is indispensable for oocyte activation and meiotic exit, while exogenous δ isoforms can rescue γ deficiency, demonstrating catalytic sufficiency but physiological non-redundancy [53,54]. At anaphase onset, CaMKII promotes Anaphase Promoting Complex/Cyclosome APC/C activation and spindle depolymerization, coupling Ca^2+^ oscillations to timely chromosome segregation [55]. These findings highlight nuclear CaMKII as a versatile regulator of the cell cycle, balancing proliferation, checkpoint enforcement, and developmental progression. Yet, most evidence remains model-specific and frequently derived from inhibitor studies; rigorous isoform-resolved analyses are needed to define its precise contributions across tissues. These isoform-specific mechanisms of cell-cycle regulation are depicted in Figure 5, showing how CaMKIIδB enforces both mitotic entry and stress checkpoints, while CaMKIIα promotes G1/S transition via Tiam1–Rac1 signaling.

### 3.5. Lineage Specification and Developmental Patterning

CaMKII isoforms function as developmentally programmed regulators that imprint lineage fate and morphogenetic identity. Within the nucleus, isoform-specific entry and substrate selection translate Ca^2+^ dynamics into long-lived transcriptional programs that couple signaling context to developmental trajectories [56].

Nervous system. During neuronal maturation, CaMKIIα and CaMKIIβ expression rises sharply and becomes enriched in excitatory circuits, coincident with synaptogenesis and circuit stabilization [57,58]. Perturbation studies demonstrate that CaMKII activity is instructive for dendritic and spine maturation, rather than a secondary correlate [59]. In dopaminergic neurons, nuclear entry of CaMKIIδ3 is regulated by PP1-dependent dephosphorylation at Ser332, unmasking its NLS and enabling translocation. Once inside, δ3 enhances BDNF transcription, promoting neurite extension and survival—linking D_2_-receptor activity to nuclear gene programs that drive arborization [15].

Heart development. The δ isoform landscape undergoes developmental remodeling via regulated alternative splicing (ASF/SF2), shifting the balance among splice variants during postnatal maturation [36,60]. Nuclear-retained δB supports progenitor survival and cardiogenic commitment by up-regulating MEF2C and accelerating lineage maturation, whereas mis-splicing or loss of δB disrupts these trajectories [8,37,61].

Skeletal muscle. CaMKII contributes to myogenic specification by phosphorylating class IIa HDACs, particularly HDAC4. This phosphorylation creates 14-3-3 docking sites, promoting nuclear export and relieving MEF2 repression. Gain-of-function experiments confirm that HDAC4 phosphorylation at Ser467 correlates with induction of oxidative and myogenic gene programs [62,63,64]. Beyond transcription-factor modulation, δ isoforms also phosphorylate histone H3 at Ser10, linking calcium flux to chromatin accessibility and long-lived transcriptional potential in cardiomyocytes [9].

Human genetics. Pathogenic variants of *CAMK2G* establish a causal role in neurodevelopmental disorders. The p.Arg292Pro gain-of-function mutation enhances kinase activity while perturbing nuclear targeting of the NLS-containing *CAMK2G* isoform, impairing neuronal migration and maturation, and leading to intellectual disability [32]. Together with *CAMK2A/B* cohorts linked to developmental syndromes, these findings reinforce the concept that nuclear CaMKII isoforms act as lineage coders—translating transient Ca^2+^ fluctuations into durable transcriptional states that define cellular identity and developmental potential [33].

## 4. Nuclear CaMKII in Pathology: Isoform-Specific Mislocalization, Misregulation, and Molecular Rewiring

Nuclear CaMKII isoforms act as transcriptional architects in health, but in disease the same circuitry is retimed, mislocalized, or over-sustained, converting adaptation into pathology. Pathogenic remodeling rarely invents new pathways; rather, it repurposes established ones: premature or persistent nuclear import, substrates phosphorylated out of context, and isoform switching that disrupts developmental targeting. In the sections that follow, we highlight representative axes: CaMKIIγ–driven nuclear signaling in cancer and therapy resistance; developmental and cardiac mis-specification driven by δ-isoform partitioning (δB/δC/δ9); and epigenetic “memory” wherein class IIa HDAC export and histone marking—and, in diabetes, O-GlcNAc–dependent CaMKII autonomy—stabilize maladaptive transcriptional states [5,6,7,8,9,19,27,33,35,36,60,65].

### 4.1. Transcriptional Oncoprotein: Nuclear CaMKII in Cancer

Among CaMKII isoforms, the γ isoform has the strongest evidence base as a nuclear regulator of oncogenic transcriptional programs. In chronic myeloid leukemia (CML) stem/progenitor cells, CaMKIIγ activity sustains survival pathways including NF-κB and Wnt/β-catenin; genetic knockdown or pharmacologic inhibition with berbamine suppresses leukemic “stemness” and prolongs survival in preclinical models [66]. In T-cell acute lymphoblastic leukemia, CaMKIIγ stabilizes c-Myc through Ser62 phosphorylation and phosphorylates FOXO3a, promoting proliferation, clonogenicity, and tumor growth [67]. In multiple myeloma, CaMKIIγ is frequently up-regulated and maintains STAT3 and ERK signaling; gain- and loss-of-function studies demonstrate dependence on CaMKIIγ, and berbamine analogs show preclinical efficacy against CaMKIIγ-positive xenografts [68]. In glioblastoma (GB xenografts), CaMKII activity—including but not limited to CaMKIIγ—supports stem-like cell maintenance, and CaMKII inhibition reduces stemness and growth [69]. While these findings establish CaMKIIγ as a critical nuclear integrator of oncogenic transcription, it is important to note that inhibitors such as berbamine have multiple targets, and isoform-resolved mechanisms remain under active investigation.

### 4.2. Developmental Mis-Specification: CaMKII in Neurocardiac Lineage Programs

During development, the consequence of nuclear CaMKII dysregulation is not uncontrolled proliferation but mis-specification of cell fate. Nuclear CaMKII isoforms act as transcriptional interpreters of differentiation, and when misrouted, they destabilize lineage programs.

Nervous system. In developing excitatory neurons, CaMKIIγ functions as a cyto-nuclear shuttle that ferries Ca^2+^/calmodulin into the nucleus, enabling CREB phosphorylation and the induction of immediate early genes required for plasticity [12,19]. Deletion of *CAMK2G* or disruption of its CaM-trapping capacity abolishes this transcriptional coupling and impairs LTP and memory formation in vivo. Human genetics reinforce this axis: the de novo *CAMK2G* p.Arg292Pro mutation yields a gain-of-function with aberrant nuclear targeting and impaired neuronal maturation, causing intellectual disability [32,33]. In dopaminergic neurons, PP1-dependent dephosphorylation unmasks the δ3 NLS, allowing nuclear entry where CaMKIIδ3 drives BDNF transcription and promotes neurite extension [15].

Heart. Postnatal cardiac maturation requires an ASF/SF2-directed splicing program that balances the nuclear δB and cytosolic δC splice variants [60]. This ensures proper partitioning between transcriptional regulation and excitation–contraction coupling. Perturbation of this balance is maladaptive: persistent δB signaling is sufficient to drive hypertrophy and dilated cardiomyopathy [8], whereas excess δC promotes calcium mishandling and contractile deterioration [35,36,61].

Oocyte/early embryo. In mammalian eggs, CaMKIIγ is indispensable for metaphase-II exit and egg activation; δ isoforms can substitute experimentally, whereas CaMKI/IV require forced activation, underscoring CaMKII’s central role in decoding fertilization calcium transients into embryonic cell-cycle progression [53,54].

### 4.3. Cardiac Bifurcation: Nuclear CaMKII at the Edge of Adaptation and Failure

In adult myocardium, outcomes critically depend on δ-isoform partitioning. CaMKIIδB contains a nuclear localization sequence and preferentially engages transcriptional programs, whereas CaMKIIδC remains largely cytosolic and couples to excitation–contraction (EC) substrates [35,36]. A third splice variant, δ9, augments nuclear inflammatory signaling via the NF-κB axis in cardiomyocytes [7]. These isoform biases determine whether stress responses remain adaptive or progress toward failure.

Calcium handling and arrhythmia. CaMKII activity increases RyR2-S2814 phosphorylation, promoting diastolic SR Ca^2+^ leak and arrhythmia susceptibility (transgenic mouse models); δC overexpression in vivo accelerates ventricular dilation and mortality [70,71,72]. CaMKII also facilitates Cav1.2 current through phosphorylation at S1512/S1570, enhancing Ca^2+^ influx and EC gain [73,74,75].

Inflammatory signaling. After ischemia/reperfusion, CaMKIIδ rapidly activates NF-κB in vivo, driving pro-inflammatory transcription [6]. In parallel, NF-κB can repress KChIP2 (KCNIP2), a determinant of I_to and repolarization reserve, linking CaMKII–NF-κB signaling to pro-arrhythmic electrical remodeling [76]. δ9 appears to be the principal isoform driving this inflammatory axis [7].

Transcriptional and epigenetic regulation. Nuclear δB phosphorylates HDAC4 (Ser467/Ser632), promoting 14-3-3 docking and nuclear export, thereby derepressing MEF2; it also phosphorylates HSF1, inducing HSP70 and cytoprotection under stress [25,39,77]. However, persistent δB signaling is sufficient to drive hypertrophy and dilated cardiomyopathy in transgenic mice, highlighting a shift from adaptive to maladaptive remodeling over time [8].

Splicing regulation. The ASF/SF2 (SRSF1)-directed postnatal splicing program governs δB/δC balance and CaMKIIδ localization; disruption mislocalizes δ isoforms and impairs Ca^2+^ handling in mice [60]. Additional regulators such as RBFOX1/2 also influence *CAMK2D* splicing, but patient-level evidence for a consistent δB→δC shift in ischemic cardiomyopathy remains limited [36].

### 4.4. Chemoresistance and Transcriptional Escape: Nuclear CaMKII in Therapy Failure

A recurrent hallmark of chemoresistance is transcriptional reprogramming—upregulation of survival, efflux, and stress-response pathways that blunt cytotoxic efficacy. Among CaMKII isoforms, CaMKIIγ is the most consistently implicated at this interface. In leukemic and myeloma systems, CaMKIIγ sustains NF-κB, β-catenin, STAT3, and ERK signaling, reinforcing stem-like properties and creating a permissive environment for therapy escape [66,67,78].

Experimental evidence. Pharmacologic or genetic CaMKII inhibition sensitizes cancer cells to standard therapies. In breast cancer (human breast cancer cell line MCF-7), the small-molecule inhibitor KN-93 enhances cytotoxicity when combined with doxorubicin, ionizing radiation, or photodynamic therapy. In melanoma, KN-93 restores TRAIL sensitivity via downregulation of c-FLIP [78,79]. More recently, Pak1 was shown to directly phosphorylate and activate CaMKII in breast cancer, and dual inhibition of Pak1 and CaMKII synergistically reduced proliferation, migration, invasion, and xenograft growth in triple-negative and Her2+ models [80]. These data underscore the role of CaMKIIγ as a transcriptional oncoprotein that preserves survival networks under therapeutic stress, and further reveal an upstream Pak1–CaMKII axis as a potential co-target for intervention.

Downstream resistance nodes. Several CaMKII-linked pathways converge on established mediators of multidrug resistance. For example, HIF-1α induces MDR1/P-glycoprotein expression, and its inhibition reverses resistance by restoring intracellular doxorubicin accumulation in colon cancer models [81]. Such findings place CaMKIIγ within a broader transcriptional axis that coordinates hypoxia responses, drug efflux, and survival.

Therapeutic implications. Tool inhibitors such as KN-93/KN-62 provide proof-of-concept that dampening CaMKII can re-sensitize tumors, but their significant off-target actions—direct interactions with voltage-gated Ca^2+^, K^+^, and Na^+^ channels, as well as calmodulin binding—preclude translational application [82,83,84]. A more selective strategy is to interrupt nuclear-directed CaMKIIγ functions. Disrupting calmodulin trapping and CaMKIIγ-mediated CaM shuttling to the nucleus—critical steps that couple surface Ca^2+^ signals to CREB-driven transcription—represents a promising direction [78,85]. Such strategic uncoupling of nuclear CaMKIIγ aims to blunt transcriptional persistence while preserving physiological CaMKII signaling in excitable tissues [86].

### 4.5. Epigenetic Miswiring and Transcriptional Memory in Chronic Disease

Chronic diseases are often sustained not by acute insults but by persistent transcriptional states. In cardiomyocytes, nuclear CaMKIIδ interfaces with chromatin regulators: phosphorylation of class IIa HDACs—particularly HDAC4 at Ser467/Ser632—creates 14-3-3 docking sites, promotes nuclear export, and derepresses MEF2 target genes. Under hemodynamic stress, CaMKIIδ also phosphorylates histone H3 at Ser10, consistent with stress-induced chromatin remodeling [5,9,77]. Repeated activation of these mechanisms can convert transient Ca^2+^ elevations into durable shifts in gene accessibility and transcriptional output.

Metabolic memory via O-GlcNAc–CaMKII. In diabetes, hyperglycemia induces O-GlcNAcylation of CaMKII at Ser279/Ser280, generating autonomous kinase activity that persists after Ca^2+^ declines. This modification promotes arrhythmogenic remodeling, and Ser280 O-GlcNAcylation is required for hyperglycemia-induced arrhythmia susceptibility in mice [27,65]. Such post-translational marks create biochemical inertia, maintaining CaMKII-dependent outputs even when the initiating insult wanes.

Endothelium and flow cues. In vascular endothelium, laminar shear stress activates a Ca^2+^/calmodulin–CaMKII–HDAC5 pathway, driving HDAC5 nuclear export and inducing a MEF2→KLF2/eNOS transcriptional program that is fundamentally atheroprotective. Disturbed flow blunts this axis and stabilizes a pro-inflammatory state, thereby priming vascular pathology [87,88].

Skeletal muscle. CaMK-dependent phosphorylation of HDAC4/5 promotes their nuclear export, relieving MEF2 repression and activating PGC-1α–dependent metabolic programs that favor oxidative phenotype and mitochondrial biogenesis [65,89]. While this mechanism is well established in exercise and differentiation, its direct contribution to sarcopenia or systemic insulin resistance remains hypothetical and requires isoform-resolved evidence.

**Figure 6:**
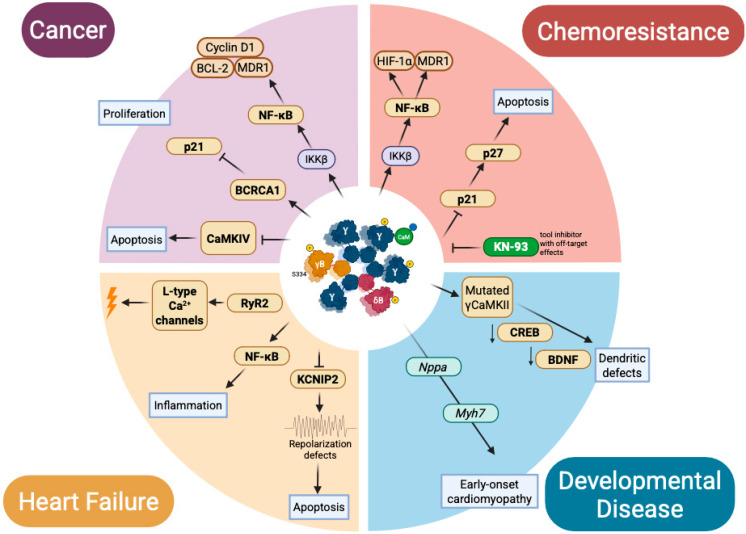
Nuclear CaMKII in pathology: context-specific misregulation across disease domains. Schematic representation of isoform- and pathway-specific consequences of nuclear CaMKII dysfunction. In cancer, CaMKIIγ sustains proliferation and survival via NF-κB, BCL-2, cyclin D1, MDR1, and BRCA1 signaling. In chemoresistance, CaMKIIγ maintains pro-survival transcriptional programs (NF-κB, HIF-1α, MDR1) and suppresses apoptosis, while tool inhibitors such as KN-93 partially restore chemosensitivity. In heart failure, CaMKIIδ isoforms drive maladaptive remodeling via RyR2 hyperphosphorylation, Ca^2+^ mishandling, NF-κB activation, KCNIP2 repression, and repolarization defects, promoting inflammation and apoptosis. In developmental disease, pathogenic *CAMK2G* mutations (CaMKIIγ) disrupt CREB/BDNF signaling and dendritic development, while CaMKIIδ mis-splicing alters cardiac gene programs (e.g., Nppa, Myh7), leading to early-onset cardiomyopathy. Collectively, these mislocalized or mistimed nuclear CaMKII signals convert adaptive decoding into maladaptive transcriptional reprogramming.

Taken together, these observations position nuclear CaMKII isoforms as central nodes in pathological rewiring across cancer, chemoresistance, heart failure, and developmental disorders. A schematic overview of these isoform-specific mechanisms and disease outcomes is presented in Figure 6.

## 5. Unresolved Dimensions and Strategic Directions: Charting the Nuclear Landscape of CaMKII

The story of nuclear CaMKII is incomplete—not for lack of relevance but because the available evidence only sketches its complexity. What is known establishes its physiological and pathological importance; what is unknown defines the frontier. The nucleus is not merely another subcellular location—it is a logic gate where CaMKII isoforms act as timekeepers, transcriptional editors, and memory encoders. Several unresolved dimensions now demand systematic exploration.

First, the nuclear substrate map remains fragmentary. Established targets include class IIa HDACs—particularly HDAC4—where phosphorylation at Ser467/Ser632 promotes 14-3-3 binding and nuclear export, derepressing MEF2-driven transcriptional programs. CaMKIIδB also phosphorylates HSF1 to induce iHSP70 and cytoprotection, while phosphorylation of the E3 ligase Pirh2 stabilizes p53 by limiting its degradation [5,25,39,52]. Yet, systematic nuclear phosphoproteomics under controlled Ca^2+^ and redox conditions is still required to define cohesive substrate clusters.

Second, isoform-specific duality requires resolution. CaMKIIγ functions as a courier, shuttling Ca^2+^/calmodulin into the nucleus to couple surface activity with CREB-driven gene expression. In contrast, δB behaves as a canonical nuclear kinase, editing transcriptional tone through chromatin regulators and stress transcription factors. This division of labor—γ as coordinator, δB as editor—may represent a sequential logic wherein γ primes nuclear access and δB establishes durable transcriptional change [12,28].

Third, isoform expression itself is a regulated signal. In the heart, postnatal splicing controlled by ASF/SF2 rebalances CaMKIIδ variants: δB (nuclear) versus δC (cytosolic). This switch dictates whether CaMKII engages transcriptional programs or excitation–contraction substrates, and its disruption deranges calcium handling and remodeling [36,60]. Whether this splicing axis can be therapeutically tuned remains an open challenge.

Fourth, therapeutic targeting requires topological precision. Class-wide inhibitors such as KN-93 remain mechanistically informative but clinically blunt. δB’s nuclear localization sequence, for example, can re-route other isoforms into nuclei, raising the possibility of selectively disrupting importin interactions or docking modules rather than globally silencing kinase activity [27,70]. Beyond class-wide tools such as KN-93, repurposing screens are beginning to surface clinically realistic CaMKII antagonists. Using a high-performance CaMKII activity reporter (CaMKAR) to interrogate 4,475 approved compounds, Reyes-Gaido et al. identified ruxolitinib as a potent CaMKII inhibitor at clinically relevant exposures; the drug suppressed CaMKII-dependent arrhythmogenesis across cultured cardiomyocytes and mouse/patient-derived models and, at cardioprotective doses, did not impair performance on standard cognitive assays [90]. These data nominate ruxolitinib—despite its primary JAK1/2 activity—as a pragmatic short-term CaMKII antagonist for cardiac indications and an orthogonal probe to test CaMKII-dependent mechanisms in vivo. In parallel, substrate-competitive peptide inhibitors such as AIP (autocamtide-2–related inhibitory peptide) and AC3-I afford high on-target efficacy with defined subcellular footprints, but their delivery and stability constraints have confined them largely to preclinical use. Together, these approaches reinforce that topological precision—biasing nuclear import/export or disrupting isoform-specific docking—may outperform global catalytic silencing for therapeutic intent [90].

Finally, molecular memory mechanisms remain underexplored. Oxidation of Met281/282 generates Ca^2+^/CaM-independent activity, while O-GlcNAcylation at Ser280 under hyperglycemia produces autonomous kinase function linked to arrhythmogenic remodeling [27,82,91]. Whether these modifications alter nuclear targeting or substrate specificity is unresolved, but they suggest that CaMKII encodes not just immediate calcium spikes but long-lived transcriptional states.

## 6. Conclusions

Nuclear CaMKII isoforms emerge as spatiotemporal regulators that convert transient calcium signals into durable transcriptional outcomes. Rather than functioning as redundant kinases, they exhibit isoform-specific logic: CaMKIIγ acts as a cytonuclear courier coupling surface activity with CREB-driven gene programs, δB functions as a chromatin-embedded kinase shaping transcriptional tone, and δC/δ9 operate as cytosolic–nuclear variants that tip the balance between adaptation and pathology. These roles extend beyond physiology into disease, where mistimed import, isoform imbalance, or persistent post-translational modifications repurpose adaptive signaling into maladaptive remodeling, cancer progression, or therapy resistance. The conceptual advance is clear: localization is function. In CaMKII biology, nuclear access is not an incidental property but a decisive determinant of cellular fate. Yet critical questions remain unanswered: Which nuclear substrates define the core transcriptional code? How do redox and metabolic modifications reshape nuclear specificity? Can isoform-resolved targeting reprogram nuclear CaMKII without undermining physiological decoding in excitable tissues? Resolving these dimensions will demand integrated strategies—structural mapping of splice isoforms, nuclear phosphoproteomics, and in vivo models linking CaMKII splicing to lineage and disease. By integrating experimental advances up to 2024, this review establishes a unified conceptual framework in which nuclear CaMKII isoforms function as programmable switches between adaptive transcription and persistent maladaptive gene expression. Ultimately, decoding the nuclear logic of CaMKII offers more than mechanistic clarity: it frames therapeutic opportunities to interrupt maladaptive transcriptional memory while preserving the physiological encoding of experience and stress.

## Data Availability

No new data were created or analyzed in this study. Data sharing is not applicable to this article.

## Abbreviations

The following abbreviations are used in this manuscript:

APC/C	Anaphase-Promoting Complex/Cyclosome
Arc	Activity-regulated cytoskeleton-associated protein
ASF/SF2 (SRSF1)	Alternative Splicing Factor/Splicing Factor 2 (Serine/arginine-rich splicing factor 1)
BDNF	Brain-Derived Neurotrophic Factor
CaM	Calmodulin
CaMK	Ca^2+^/calmodulin-dependent protein kinase
CaMKII	Ca^2+^/calmodulin-dependent protein kinase II
CDK	Cyclin-Dependent Kinase
CBP	CREB-Binding Protein
CML	Chronic Myeloid Leukemia
CREB	cAMP Response Element-Binding protein
DCM	Dilated Cardiomyopathy
EC	Excitation–Contraction
eNOS	Endothelial Nitric Oxide Synthase
ERK	Extracellular signal-Regulated Kinase
FOXO3a	Forkhead Box O3a
GB	Glioblastoma
GIT1	G-protein-coupled Receptor Kinase-Interacting Protein 1
HDAC	Histone Deacetylase
HIF-1α	Hypoxia-Inducible Factor 1-alpha
HSF1	Heat Shock Factor 1
HSP70	Heat Shock Protein 70
IKKβ	IκB Kinase beta
iHSP70	Inducible Heat Shock Protein 70
KLF2	Krüppel-Like Factor 2
KN-93/KN-62	CaMKII pharmacological inhibitor
KChIP2	Kv Channel-Interacting Protein 2
MEF2	Myocyte Enhancer Factor 2
MDR1	Multidrug Resistance Protein 1 (P-glycoprotein)
NF-κB	Nuclear Factor kappa-light-chain-enhancer of activated B cells
NES	Nuclear Export Signal
NLS	Nuclear Localization Signal
NSCLC	Non-Small Cell Lung Cancer
O-GlcNAc	O-linked β-N-acetylglucosamine
PGC-1α	Peroxisome proliferator-activated receptor Gamma Coactivator 1-alpha
Pirh2	p53-induced protein with a RING-H2 domain (E3 ubiquitin ligase)
PKD	Protein Kinase D
PP1	Protein Phosphatase 1
RyR2	Ryanodine Receptor 2
Ser/Thr	Serine/Threonine
SR	Splicing Regulator
SR Ca^2+^	Sarcoplasmic Reticulum Calcium
STAT3	Signal Transducer and Activator of Transcription 3
T-ALL	T-cell Acute Lymphoblastic Leukemia
TAK1	Transforming growth factor-β-Activated Kinase 1
Tiam1	T-lymphoma invasion and metastasis-inducing protein 1
TRAIL	TNF-related apoptosis-inducing ligand
NFAT	Nuclear Factor of Activated T-cells
NLK	Nemo-Like Kinase
δB/δC/δ9	CaMKIIδ splice variants (nuclear B, cytosolic C, variant 9)
